# Taking a Shot: The Impact of Information Frames and Channels on Vaccination Willingness in a Pandemic

**DOI:** 10.3390/vaccines11010137

**Published:** 2023-01-06

**Authors:** Lilian O. Ademu, Jingjing Gao, Janine Rangel de Assis, Aanuoluwapo Uduebor, Ojonoka Atawodi

**Affiliations:** 1 Public Policy Program, College of Arts and Sciences, University of North Carolina at Charlotte, Charlotte, NC 28262, USA; 2 Texas A&M AgriLife Center in El Paso, Texas A&M University, El Paso, TX 79927, USA; 3 Department of Computer Science, College of Arts and Sciences, University of Southern Mississippi, Hattiesburg, MS 39406, USA

**Keywords:** vaccine skepticism, global pandemic, health communication, framing theory, survey experiments

## Abstract

The reluctance of people to receive safe and recommended available vaccines is a well-documented public health challenge. As information and communication technologies evolve, this challenge gets more complex and even harder to manage during complex public health situations. In this experimental study, we examine the relationship between vaccine information frames (with scientific information vs. without scientific information) and channels (through government vs. religious organizations) and vaccination willingness in the U.S. in the context of a pandemic. Additionally, we evaluate the interaction between vaccine skepticism, vaccine information frames, and vaccine information channels on vaccination willingness. This experimental study uses data from Amazon Mechanical Turk (MTURK) to evaluate the relationships between vaccine skepticism, vaccine information frames, and channels on vaccination willingness. We find that contrary to our hypothesis, a vaccine advisory framed with scientific information decreases people’s vaccination willingness compared to one framed without scientific information. Additionally, the impact of framing on vaccination willingness is conditioned on participants’ skepticism—participants who hold skepticism toward the vaccine but received information framed with scientific information score significantly higher in vaccination willingness compared to participants who do not hold skepticism toward a vaccine. The results suggest that the factors impacting vaccination willingness are complex and nuanced. Thus, policymakers should be more strategic with the delivery of vaccination information, especially during complex health crises.

## 1. Introduction

Before the COVID-19 global pandemic, the reluctance of people to receive safe and recommended available vaccines was already a public health issue worldwide [1]. Historically, government failures and misdeeds on some public health issues have led to citizens’ alienation and distrust, predisposing people to misinformation and conspiratorial thinking [2]. These issues are further exacerbated by media framing of issues along ideological divides [3,4]. During the 2021 pandemic, the US Surgeon General released an advisory on building a healthier communication and information environment. The report states that people spreading misinformation may not necessarily be trying to misinform but trying to make sense of conflicting information or seeking answers to sincere questions (https://www.hhs.gov/sites/default/files/surgeon-general-misinformation-advisory.pdf; accessed on 2 December 2022).

The concerns associated with the adverse effects of vaccination have lined the historical timeline of vaccination programs globally. From the development of the first vaccine by Edward Jenner in 1796 to the COVID-19 vaccine in 2020, vaccine skepticism has been an issue of public health concern. A study found that despite varying reasons for vaccine refusal across cultures and contexts, the main concerns for vaccine refusal were fears about safety issues and adverse side effects [5]. The impact of skepticism about the safety and efficacy of vaccines can be found in multiple scientific studies [6]. People who perceive vaccines as unsafe are less likely to vaccinate both themselves and their children against vaccine-preventable diseases [7,8]. The Wakefield et al. publication claiming a relationship between the MMR vaccine and childhood autism led to many Americans holding skeptical opinions about the safety of childhood vaccines [9,10]. Social trust facilitates the collective action required to attain adequate levels of vaccination in a population [11]. This is even more salient during extreme health crises when there is little knowledge about a disease, and the government must implement certain health countermeasures amidst an overabundance of health messaging and misinformation [12,13,14].

How health information is framed and channeled plays a significant role in improving public trust and thus the effectiveness of health interventions. Past studies demonstrate that the frames and channels of health information significantly impact citizens’ trust and adherence to public health advisories [15,16]. Message framing has been extensively studied in human papillomavirus vaccination, HIV/AIDS interventions, and Ebola virus advisories [17,18,19]. Overall, these studies demonstrate how strategic framing of health information could effectively build target populations’ trust in a vaccine intervention. For instance, a recent study suggests that presenting individuals with information specifically about the safety of COVID-19 vaccines increases their willingness to take the vaccine [10]. Additionally, channels of health information also matter in implementing health interventions. Current research also suggests that different channels of media communication impact people’s acceptance of the COVID-19 vaccine [20].

The frames and channels of health communication are salient when one considers the digital context in which public health information is communicated today. The plethora of multiple sources of health information and misinformation in circulation makes it more challenging for the successful communication of public health interventions and programs. Furthermore, why health messages are accepted or rejected has been linked to prior attitudes citizens may have about a public health issue [21]. The COVID-19 pandemic has been riddled with many conspiracy theories and misinformation concerning the recommended vaccines. Studies have found that the backfiring of health information is more likely among those with prior negative attitudes toward vaccination [22].

In this study, we explore the following questions: in complex public health situations like a pandemic, do the frames and channels of health information regarding a public health intervention matter for vaccination willingness? How does vaccine skepticism interact with the frames and channels to impact vaccination willingness during a global pandemic?

### Theory and Hypothesis

This study builds upon health communication theories while focusing on the effect of vaccine information frames and channels on vaccination willingness during a pandemic. We examine how vaccine information frames (with scientific information vs. without scientific information) and channels (through government vs. religious organizations) impact citizens’ vaccination willingness in the United States. Framing theory is useful in understanding the decisions people make concerning their health, particularly why they may choose to take a vaccine shot or not [23,24]. The effect of message framing has been studied in different domains of public health. Research in these domains indicates that the way a health intervention is framed is associated with people’s trust and health risk perceptions [25,26,27,28]. This concept has been used to design messages to nudge individuals to make the desired health decisions [29]. A large body of empirical research on message framing in the context of vaccination accumulated over the past decade shows the importance of framing effects on people’s vaccination [30].

Research indicates that while some messaging strategies are effective in reducing vaccine skepticism, some have backfired [8], so the design and communication of vaccine messages or vaccine framing are critical in vaccination interventions [31]. A recent study demonstrates the impact of message framing in changing people’s willingness to take up any of the available COVID-19 vaccines [15]. Furthermore, public health experts argue that providing adequate background information and contexts through thematic framing has the potential to improve public understanding of systemic disease risk factors and encourage public adherence to health interventions [32]. Our study contributes to the literature by using thematic framing effects as opposed to equivalency framing effects that use positive or negative information to frame messages [33,34]. Based on the literature and our study goal, we examine the impact of vaccination advisory frames (with scientific information vs. without scientific information) on vaccination willingness. Our first hypothesis is:

**H1.**  *A vaccination health advisory framed with scientific information would increase citizens’ willingness to take the vaccine*.

Beyond framing, the channels used for communicating public health interventions also have a profound impact on citizens’ trust and adherence to health advisories [35]. Due to its importance for public health, the WHO has emphasized the use of channels that citizens trust to communicate and implement health programs (https://www.who.int/about/communications/accessible/identify-effective-channels; accessed on 20 December 2022). In the United States, instances where this phenomenon impacted people’s preventive and reactive healthcare decisions, have been well studied [36]. The impact of channels used in the communication of health interventions has been demonstrated in the uptake of vaccination programs like HPV, measles, and important childhood vaccines [37]. A recent study demonstrates how different channels of media communication impact people’s acceptance of the COVID-19 vaccine [20].

In many parts of the world, the acceptance of public health interventions and their penetration over time has also been influenced by the channels used to transmit health information. Scholars have investigated the impact of the religiousness of the channels on citizens’ trust and adherence to health advisories. Some of these studies indicate that citizens’ religious predispositions are associated with their health decisions [38]. Another study suggests that citizens are more likely to trust and adhere to COVID-19 health communications channeled through religious organizations than the government [16].

Other studies show that when faced with hardship, people turn to their faith for support [39]. This is even more salient during extreme health crises when people are more skeptical about public health institutions and health [40,41,42]. Past studies have emphasized the importance of partnering with religious or faith-based institutions to encourage citizens’ adherence to public health advisories, especially during a public health crisis or shortage of healthcare resources [43,44]. Our second hypothesis thus is:

**H2.**  *A vaccination health advisory from a religious organization would increase citizens’ willingness to take the vaccine more than one from the government*.

Vaccine skepticism plays an important role in public health since people who perceive vaccines as unsafe are less likely to decide to vaccinate both themselves and their children against vaccine-preventable diseases [45,46,47]. Studies on vaccine skepticism suggest that the framing of health communication has implications for vaccination acceptance among groups that are highly skeptical about vaccination [6,48]. This study also evaluates the moderating roles of vaccine information frames and channels on the relationship between skepticism and vaccination willingness. Our corresponding hypotheses of the interaction effects between skepticism, and vaccine information frames and channels, on vaccination willingness, are:

**H3a.**  *There is an interaction between skepticism and a vaccine advisory framed with science on vaccination willingness*.

**H3b.**  *There is an interaction between skepticism and vaccine information channeled through a religious organization on vaccination willingness*.

Additionally, individuals with skepticism towards a vaccine may respond more positively to vaccination advisories with more scientific information and background about the development and safety of a vaccine. Providing more scientific information about the development and efficacy of vaccines is a way to frame a vaccination health advisory to encourage vaccination willingness. Our fourth and last hypothesis thus is:

**H4.**  *For those skeptical about a vaccine, a vaccination health advisory framed with scientific information would increase their willingness to take the vaccine*.

## 2. Materials and Methods

### 2.1. Research Design

A 2 × 2 between-subjects survey experiment that manipulated vignettes presented to research participants was used to test the hypotheses [49,50]. Participants were administered treatments where the channel and framing of the health information in each vignette were randomly assigned. We apply contextual realism in the designing of the vignettes; participants were presented with a vaccination advisory that closely resembles the health advisories that have been released by the CDC or FDA during the COVID-19 pandemic. Hence, respondents are likely to view the health information as one they may realistically see and form an opinion on taking the vaccination or not.

The experimental vignettes (Table A1 in the Appendix A) were administered to 500 members of the Amazon Mechanical Turk (MTURK) community in the spring of 2022 as part of an online omnibus study. Qualtrics, the survey platform used for the experiment, randomly assigned respondents to one of the four conditions. This random assignment was checked by constructing a balanced table of demographic characteristics (Table 1). Though there have been criticisms of the use of MTURK survey samples for research [51,52,53], research still suggests that MTURK samples are still useful for research if conditions such as attention checks and English language fluency are considered for internal validity and construct validity [54,55].

The dependent variable—citizens’ willingness to take the vaccine after reading the health advisory was used as the outcome to test the hypotheses. This was measured on a five-point Likert-type scale (1 = very unlikely, 2 = somewhat unlikely, 3 = neither likely nor unlikely, 4 = somewhat likely, and 5 = very likely). The key independent variables were the frames and channels of health information. The channel of health information was coded into government or a religious organization, while the framing was either with scientific information (“science”) or without scientific information (“no science”) (Table A1). A question capturing each participant’s skepticism of the vaccine was also asked (Table A2). This was measured on a three-point Likert-type scale (1 = Yes, 2 = No, and 3 = I’d rather not answer). The experimental vignettes were followed by demographic questions.

Attention or memory checks were the final questions administered to check if respondents paid attention to the questions asked [49,50]. The attention check question asked the number of months it took to develop a vaccine for the virus responsible for the hypothetical pandemic. In the robustness analysis, we use the sample of only the respondents who passed the attention check [49,50]. Furthermore, browser and URL blockers were activated to ensure that only individuals residing in the United States participated in the survey. The survey took an average of 25 min to complete and those who completed the survey were paid two dollars and fifty cents for participating in the survey. In the following statistical analysis, we conduct an intent-to-treat analysis on the entire sample, which should be the most conservative way to analyze the data [54].

### 2.2. Statistical Analysis

We use ordinary least squares (OLS) models to test our hypotheses. OLS models are unbiased even if it technically violates the Gauss–Markov assumptions, which only affect tests of significance. Furthermore, OLS modeling is also preferred for ease of interpretation [49,56]. We also ran the model as an ordinal logistic regression, and the sign and statistical significance of the parameters is similar to the linear model. The models include interactions between the frame and channel of communication and each respondent’s skepticism towards the vaccine (a 0/1 dichotomous variable for skepticism). By adopting multivariate models, we provide the most conservative analyses to test our hypotheses [16,50]. The first model, in which the baseline categories are “no science” and “government”, answers our research questions. In a second model, we use the baseline category of “government, no science” to further examine how both the frames and channels together interact with skepticism to impact people’s vaccination willingness. The socio-demographic variables included in the models are age, race, gender, educational level, political ideology, marital status, and household income. We describe the model used for each analysis below:

*Vx = β_1_(Sci × Scep) + β_2_(RO × Scep) + Dx + ε_i_* (1)

*Vx = β_1_(GovSci × Scep) + β_2_(RONoSci × Scep) + β_2_(ROSci × Scep) +Dx + ε_i_* (2)

In model 1, *Vx* is a respondent’s willingness to take the hypothetical vaccination. This was measured on a five-point Likert-type scale (1 = very unlikely, 2 = somewhat unlikely, 3 = neither likely nor unlikely, 4 = somewhat likely, and 5 = very likely). On the right-hand side of the equation, *Sci* is an indicator for treatment with a “science” frame and *Scep* is an indicator of a respondent’s skepticism towards the hypothetical vaccine. This was recorded as a 0/1 dichotomous variable for skepticism. The option “I’d rather not answer” was recorded as “NA” in the analysis. *RO* is an indicator for treatment with a religious organization as the channel of vaccination communication. The vector Dx is the set of demographic controls described above. In model 2, *GovSci* is an indicator for treatment with both a science frame and government as the channel of communication, *RONoSci* is an indicator for treatment with a religious organization as the channel of communication with a “no science” frame, and *ROSci* is an indicator for treatment with both religious organization as the channel of the vaccination communication and a “science frame”. All other indicators remain as earlier described.

## 3. Results

A total of 498 respondents completed the experimental survey. Our findings offer a nuanced view of how citizens are likely to respond to vaccination advisories during a pandemic. This is especially important because as citizens in such contexts, people are exposed to health information with different frames and from different channels.

### 3.1. Descriptive Results

The balance table, Table 1, shows that the demographics—age, gender, marital status, conservatism (political ideology), and college education—are similar across the four treatment groups. This indicates that the random assignment design of the survey experiment was successful.

Table 2 shows the descriptive statistics of some of the demographics—the control variables, used in the multivariate regression analysis. The study sample is made of mostly males (60%), white (79%), college graduates (86%), and those who are married (73%).

### 3.2. Regression Results

Model 1 in Table 3 shows that framing the health advisory with more scientific information is significantly associated with participants’ vaccination willingness (*p* < 0.05). However, the direction of effect is the opposite to our first hypothesis. Vaccine information framed with scientific information is negatively associated with citizens’ willingness to take the vaccine. Model 1 also shows that, though not statistically significant, participants are more willing to vaccinate if the health information was channeled through a religious organization. This is in tandem with our second hypothesis.

**Figure A1:**
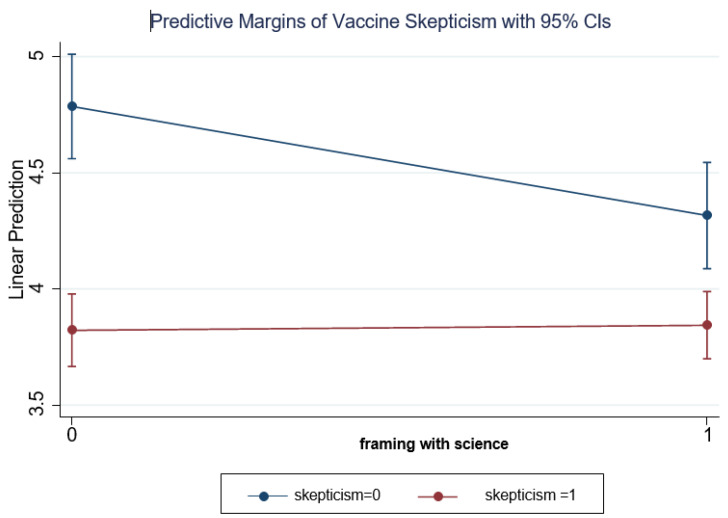
Interaction between science and skepticism.

The results also indicate that skepticism is negatively associated with participants’ vaccination willingness (*p* < 0.05), and participants who hold skepticism toward vaccines are significantly less likely to get vaccinated. For our third hypothesis, we evaluate the interaction between participants’ skepticism and the health information frame and channel. The results suggest that the interaction effect is only significant for the health information framed with science. We also find support for our fourth and last hypothesis. For those skeptical about the vaccine, a vaccination health advisory framed with scientific information significantly increases their willingness to take the vaccine (*p* < 0.05). Furthermore, this effect size is large enough to eliminate the overall negative impact of framing with science (0.492 > −0.470) on vaccination willingness. Figure A1 in the Appendix A shows this interaction between science and skepticism.

**Figure A2:**
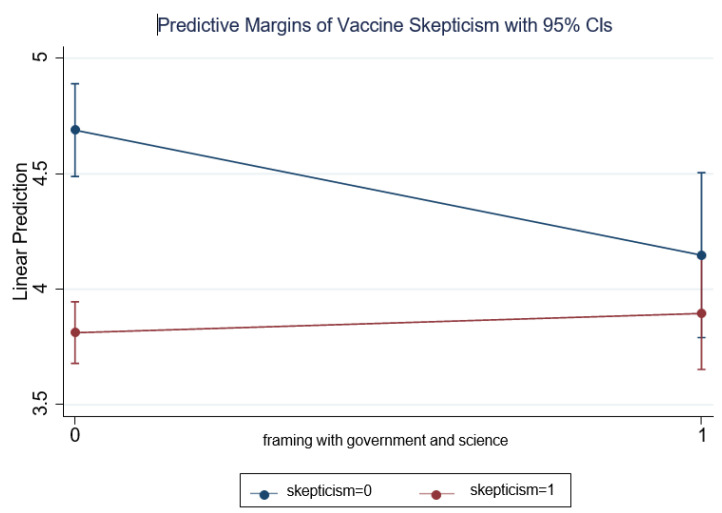
Interaction between both science and government (frame and channel) and skepticism.

The results in model 2 show the interaction between both vaccine information frames and channels on vaccination willingness. Compared to participants who received treatment of “government, no science”, those who received the treatment of “government, science” scored significantly lower (*p* < 0.05) in vaccination willingness. Overall, the results are very similar to model 1. While framing with science is significantly and negatively associated with citizens’ willingness to take a vaccine during a pandemic, the channels of health information are not. Compared to participants who received the treatment of “government, no science”, those who received the treatment of “government, science “and “religious organization, science” were less willing to take the vaccine. Those who received treatment of “religious organization, no science” were more willing to take the vaccine while those who received the treatment of “government, no science”. Figure A2 in the Appendix A shows this interaction effect between “government, science”, and skepticism.

The results also suggest that participants with a college degree (*p* < 0.05) and participants who are married (*p* < 0.05) score significantly higher in vaccination willingness compared to others. Also, people who identify as conservatives score significantly lower in vaccination willingness. Finally, these results are still robust after considering only respondents (N = 365) who passed the attention check (Table A3).

## 4. Discussion

Central to this study is the premise that the way health communication messages are channeled and framed contributes to the success of public health interventions. This is even more salient during unique contexts like a global health pandemic when consumers of health information are neither passive nor neutral receptors of public health advisories, but active constructors of risk [57].

In this study, we examine how the frames and channels of a health communication impact citizens’ willingness to take a vaccine during a global health crisis. We also examine how vaccine skepticism interacts with these frames and channels to impact people’s willingness to take a vaccine. While we find no evidence that the channel of a vaccine advisory significantly impacts citizens’ vaccination willingness, we find significant evidence of framing effects, though not in the expected direction of our hypothesis. Overall, our results suggest that the framing of vaccination advisory with scientific information negatively impacts citizens’ willingness to take up a vaccine. This finding not only provides evidence of the salience of framing in unique contexts like a global health crisis, but it also re-emphasizes the argument that the reasons behind vaccine hesitancy are complex, nuanced, and encompass more than just a knowledge deficit [3].

While we find no significant impact from the channel of health communication, past studies in both the global North and South have found significant relationships between the channels of health communication and public compliance with health advisories [16,58,59]. It can also be argued that the positive impact of the treatment with religious organizations on vaccination willingness may be a result of citizens perceiving these organizations as more objective since they are not traditionally seen as having a vested interest in promoting vaccination.

Scholars argue that in extreme public health crises, citizens may construct risks in ways that public health officials may interpret as irrational or ignorant [60,61]. Much like this study, recent research has found negative associations between science and confidence about vaccination. A recent study finds that vaccine uptake is less likely in countries with low levels of trust or confidence in science and scientists [62]. The WHO posits that the willingness to take up vaccines is a function of confidence, convenience, and complacency [63]. The confidence component of this tripartite framework results from the trust citizens have invested in the systems, institutions, and public agents, including the healthcare systems and the science that underlies health interventions [64]. In unique health situations like a pandemic, trust in science is thus an efficient heuristic shortcut for making decisions about the safety and efficacy of a vaccine [62].

A recent study by the Pew Research Center shows a sharp decline in Americans’ trust in scientists since 2019 [65]. The study highlights that the share of citizens with a great deal of confidence in scientists acting in the best interest of the public is down by 10% from 39% to 29% since the beginning of the COVID-19 pandemic. One reason for this decline in trust may be the growth in science-related populism during the COVID-19 pandemic [66]. Science-related populists argue that scientific facts are generated by elites who do not act in the best interest of the general public [67]. A recent study finds that trust in science may even lead people to make decisions counterintuitive to scientific facts. Their findings suggest that trust in science may make individuals more vulnerable to pseudoscience [68]. In another study where an experimental conjoint survey design was employed, the researchers found a negative relationship between trust in COVID-19 information from scientists and experts and those with higher levels of anti-intellectual sentiments [69]. These past studies, and our current study, further strengthen the argument that vaccination decisions by citizens are highly complex with multiple factors impacting people’s willingness to take up a vaccine.

The results of this study suggest that for those skeptical about a vaccine, a health advisory framed with scientific information increases the willingness to vaccinate. Though the interaction effect was moderately large enough to cancel the negative impact of the science framing on the willingness to take up a vaccine, this effect was not large enough to lead to an overall positive impact on skepticism. This finding agrees with a recent study that suggests that increasing trust in science was unlikely to lead to immediate improvements in complying with COVID-19 mandates [70].

The phenomenon observed in this study and past studies may be explained by bounded psychological processes associated with framing effects [71,72]. According to Chong and Druckman (2007), framing effects are dependent on a combination of factors such as “the strength and repetition of the frame, the competitive environment, and individual motivations” [73]. They argue that, in competitive contexts, how much an individual is willing to change their preexisting beliefs or opinions about an issue is a function of the strength of the opposing frame. As earlier stated, in unique contexts like a global health pandemic, individuals are exposed to a wide array of information sources as they try to make sense of their states of the world [14]. Chong and Druckman’s push-pull conceptualization of “competitive environments and individual motivations” explains why, despite the positive impact of the scientific frame on those who are skeptical, the effect was not large enough to cancel out overall skepticism [73].

This study focused on examining the impact of short-term exposure to the framing effects on citizens’ willingness to take up a vaccine. However, public opinion scholars have argued that framing effects solidify over time as people are constantly exposed to a particular framing [73]. Future studies should examine the impact of long-term exposure to different frames of public health communication in reducing the susceptibility of individuals in competing environments. This is because sustaining resistance to competitive frames may require long-term learning by individuals [74].

In addition to our experimental findings on the impact of message frames and channels on people’s vaccination decisions, some demographic characteristics were significantly associated with vaccination willingness. Individuals who identified as conservatives based on their political ideology were significantly less willing to take up the vaccine. This finding is similar to past research that finds that a more conservative ideology and evangelicalism were associated with less willingness to vaccinate for COVID-19 and positively correlated with vaccine hesitancy [75,76,77]. We also find that college graduates and those who are married were more willing to take the vaccine. These findings are also similar to past studies examining these demographic groups [78].

This study has some limitations. The most important is the non-generalizability of the findings to the total US population. For one, the study is not a representative sample as it is made up largely of married individuals (73%) and college graduates (86%). Another limitation of the study is that the study was done during an ongoing global health pandemic and at a time when the efficacy of the COVID-19 vaccine was still being debated. While it creates a good argument for mundane realism, the public health, political and social context of this period may have shaped the opinions of those recruited into the study. This opinion may hence change across different contexts and timelines. Lastly, while the sample size used in this study is quite appropriate for statistical analysis, a larger sample size would have improved the statistical power of the results. Future studies should consider using larger samples while also testing for the impact of different contexts and timelines on peoples’ vaccination decisions. This would add a lot of value to the literature on vaccine skepticism and how frames and channels impact vaccination willingness.

### Policy Implications

The policy implications of a finding like this are crucial. First, to proffer effective and efficient interventions related to vaccination willingness during a global health crisis, health policymakers have to understand the wide spectrum of factors associated with citizens’ vaccination decisions. Without a better understanding of the nuances associated with people’s health decision-making, both national and international funds allocated for programs aimed at mitigating disease during a global pandemic would remain astutely inadequate.

Secondly, this study reinforces the current debate on the war on science and expertise. While this study suggests that providing more scientific facts in a vaccination advisory could be counterproductive, it also reveals an underlying issue related to trust and skepticism. Many scholars have argued that the reasons behind vaccine hesitancy encompass more than just a knowledge deficit [3]. They argue that the underlying issue is one of trust and recommend interventions targeted at bridging the trust gaps across different groups of individuals. Health policymakers should hence focus more on building trust rather than persuading with science, especially among marginalized groups and communities. Our findings support the recommendations made by experts [3]. Frameworks for public health campaigns around vaccines should first be built around trust-building interventions that focus on transparency and justice before expertise or science.

Lastly, this study finds a negative relationship between conservative ideology and vaccination willingness. Due to increasing political polarization and populism, understanding the impact of health messaging across different political and religious ideologies is important. The COVID-19 pandemic further exposed many ideological divides which have impacted people’s willingness to comply with health mandates targeted at curtailing the spread of the COVID-19 virus [79]. Science-related populism has also led to anti-scientific ideologies [67,80]. The issues illustrated here have long-term impacts on public compliance with health advisories, especially during unique public health situations like the COVID-19 pandemic. The findings of this study suggest that public health messages designed to increase public compliance should be more strategic and consider a more nuanced and inclusive approach in their design and implementation.

## 5. Conclusions

The findings of this study suggest that the framing of health communications significantly impacts people’s vaccination decisions. Though the results suggest that a vaccination advisory framed with science negatively impacts willingness to vaccinate, for vaccine skeptics the science frame positively impacted their vaccination decisions. The findings from this study illustrate the heterogeneity in responses to health communications across populations. Further, it highlights the nuances and complexities associated with vaccination willingness. This is even more salient during unusual situations like a global pandemic when people may be more interested in looking out for institutions that they believe would act in their best interests irrespective of their expertise or intellectual pedigree [39,69].

As discussed in the Theory section, trust is at the intersection of the decisions people make when faced with multiple sources of information and choices. Numerous studies have shown that trust increases compliance with public advisories and the channels and framing of these advisories play a role in building or eroding trust [16,81,82,83]. While we find no significant impact for the channel of health communication, past studies in both the global North and South have found significant relationships between the different channels of health communication and public compliance with health advisories [16,58,59].

## Figures and Tables

**Table 1:** Balance Table.

Treatments	% Male	Average Age	% White	% Married	% Conservative	% College Graduates
Government, no science	62.9	39.72	75.81	71.8	48.39	84.68
Government, science	57.14	39.07	81.95	74.43	46.61	87.22
Religious org, no science	52.17	36.83	80	71.3	42.61	84.34
Religious org, science	67.4	38.81	80.95	75.39	38.09	88.1
N	497	497	497	497	497	497
ANOVA (*p*-value)	F= 2.26	F = 1.50	F = 0.57	F = 0.25	F = 1.07	F = 0.35
	0.08	0.21	0.64	0.86	0.36	0.79

**Table 2:** Descriptive statistics.

	**Percentage (Mean)**	**SD**
Male	0.60	0.49
Age	38.65	11.07
Married	0.73	0.44
Conservative	0.44	0.5
College graduates	0.86	0.35
White	0.79	0.4
Black	0.12	0.33

**Table 3:** Vaccine information frame, channel, and vaccination willingness.

DV = Vaccination Willingness	Model 1	Model 2
**Either frame or channel**		
Frame: *(Baseline = No science)*		
With science	−0.470 ***	
	(0.162)	
Channel: *(Baseline = Government)*		
Religious organization	0.115	
	(0.162)	
Skepticism	−0.830 ***	
	(0.171)	
Science × Skepticism	0.492 **	
	(0.227)	
Religious organization × Skepticism	−0.364	
	(0.223)	
**Both Frame and Channel:**		
*(baseline: Government, no science)*		
Government, science		−0.541 **
		(0.268)
Religious organization, no science		0.041
		(0.227)
Religious organization, science		−0.344
		(0.239)
Skepticism		−0.899 ***
		(0.196)
Government, science × Skepticism		0.624 **
		(0.268)
Religious organization, no science × Skepticism		−0.134
		(0.276)
Religious organization, science × Skepticism		0.209
		(0.281)
**Demographics**		
Age	−0.006	−0.006
	(0.004)	(0.004)
College Education	0.471 ***	0.467 ***
	(0.142)	(0.142)
Married	0.535 ***	0.530 ***
	(0.116)	(0.117)
Conservative	−0.159 *	−0.153
	(0.094)	(0.094)
Gender *(Baseline = Female)*		
Male	0.05	0.05
	(0.095)	(0.095)
Ethnicity *(Baseline= White)*		
Black or African American	0.162	0.163
	(0.135)	(0.135)
Asian	0.241	0.241
	(0.263)	(0.263)
American Indian	0.223	0.217
	(0.199)	(0.199)
Other races	−0.384	−0.395
	(0.382)	(0.384)
HH income *(Baseline: < = $5000–$49,999)*		
$50,000–$99,999	0.086	0.086
	(0.097)	(0.097)
$100,000–$199,999	−0.114	−0.126
	(0.172)	(0.172)
$200,000–> = $300,000	−0.285	−0.289
	(0.414)	(0.417)
Observations	488	488
R-squared	0.19	0.19

Note: Standard errors are in parentheses; *** *p* < 0.01, ** *p* < 0.05, * *p* < 0.1.

## Data Availability

Not applicable.

## Appendix A

**Table A1:** Experimental vignettes administered to respondents.

		Health Information Channel	
		Government	Religious Organization
**Health information Frame**	No science	Imagine that there is a global health pandemic and millions have died across the world. Vaccines were developed 12 months into the pandemic by the world’s leading pharmaceutical companies and there is a lot of information circulating on various social media platforms about the safety of the vaccine. Some state that the vaccine is dangerous and should not be taken. Below is an excerpt from a speech released by your state’s Public Health Department:“It took selfless individuals like you and I working in collaboration with both public organizations and private companies to make the vaccines available to us. While the vaccines were developed rapidly, all steps have been taken to ensure their safety and effectiveness. We recommend you get the vaccine as soon as you can to help protect yourself and others.”	Imagine that there is a global health pandemic and millions have died across the world. Vaccines were developed 12 months into the pandemic by the world’s leading pharmaceutical companies and there is a lot of information circulating on various social media platforms about the safety of the vaccine. Some state that the vaccine is dangerous and should not be taken. Below is an excerpt from a speech released by a well-respected religious organization“It took selfless individuals like you working in collaboration with both public organizations and private companies to make the vaccines available to us. While the vaccines were developed rapidly, all steps have been taken to ensure their safety and effectiveness. Upon deliberate contemplation and research, I recommend you get the vaccine as soon as you can to help protect yourself and others.”
	Science	Imagine that there is a global health pandemic and millions have died across the world. Vaccines were developed 12 months into the pandemic by the world’s leading pharmaceutical companies and there is a lot of information circulating on various social media platforms about the safety of the vaccine. Some state that the vaccine is dangerous and should not be taken. Below is an excerpt from a speech released by your state’s Public Health Department:“It took selfless individuals like you working in collaboration with both public organizations and private companies to make these vaccines available to us. While the vaccines were developed rapidly, all steps have been taken to ensure their safety and effectiveness. Our team of scientific and medical experts has evaluated scientific data gathered from numerous sources and analyzed the efficacy of the vaccines. While there is still a lot we are learning about the virus, we are committed to providing the public with the best and most up-to-date scientific research as it unfolds. We recommend you get the vaccine as soon as you can to help protect yourself and others.”	Imagine that there is a global health pandemic and millions have died across the world. Vaccines were developed 12 months into the pandemic by the world’s leading pharmaceutical companies and there is a lot of information circulating on various social media platforms about the safety of the vaccine. Some state that the vaccine is dangerous and should not be taken. Below is an excerpt from a speech released by a well-respected religious organization“It took selfless individuals like you working in collaboration with both public organizations and private companies to make the vaccines available to us. While the vaccines were developed rapidly, all steps have been taken to ensure their safety and effectiveness. A team of scientific and medical experts has evaluated scientific data gathered from numerous sources and analyzed the efficacy of the vaccines. While there is still a lot being learned about the virus, I am committed to providing you with the best and most up-to-date scientific research as it unfolds. Upon deliberate contemplation and research, I recommend you get the vaccine as soon as you can to help protect yourself and others.”

**Table A2:** Respondent’s Skepticism Toward the Vaccine.

Respondent’s Skepticism Toward the Vaccine
After reading the scenario above: Were you skeptical about the vaccine? 1. Yes 2. No 3. I’d rather not answer

**Table A3:** Vaccine information frame, channel, and vaccination willingness.

DV = Vaccination Willingness	Model 1	Model 2
**Either frame or channel**		
Frame: *(Baseline = Without science)*		
With science	−0.415 ***	
	(0.18)	
Channel: *(Baseline = Government)*		
Religious organization	0.094	
	(0.179)	
Skepticism	−0.955 ***	
	(0.201)	
Science × Skepticism	0.465 **	
	(0.227)	
Religious organization×Skepticism	−0.364	
	(0.223)	
**Both Frame and Channel:**		
*(baseline: Government, no science)*		
Government, science		−0.463 *
		(0.249)
Religious organization, no science		0.044
		(0.252)
Religious organization, science		−0.315
		(0.261)
Skepticism		−1.017 ***
		(0.211)
Government, science × Skepticism		0.581 *
		(0.314)
Religious organization, no science × Skepticism		−0.235
		(0.326)
Religious organization, science × Skepticism		0.105
		(0.323)
**Demographics**		
Age	−0.004	−0.004
	(0.005)	(0.005)
College Education	0.472 ***	0.469 ***
	(0.164)	(0.165)
Married	0.505 ***	0.502 ***
	(0.139)	(0.140)
Conservative	−0.095	−0.095
	(0.115)	(0.116)
Gender *(Baseline = Female)*		
Male	0.021	0.018
	(0.114)	(0.115)
Ethnicity *(Baseline= White)*		
Black or African American	0.131	0.137
	(0.168)	(0.169)
Asian	0.423	0.424
	(0.292)	(0.293)
American Indian	0.423	0.34
	(0.292)	(0.215)
Other races	−0.043	−0.05
	(0.428)	(0.431)
HH income *(Baseline = < = $5000–$49,999)*		
$50,000–$99,999	0.008	0.008
	(0.12)	(0.12)
$100,000–$199,999	−0.036	−0.052
	(0.189)	(0.193)
$200,000–> = $300,000	−0.048	−0.039
	(0.528)	(0.533)
Observations	357	357
R-squared	0.24	0.24

Note: Standard errors are in parentheses; *** *p* < 0.01, ** *p* < 0.05, * *p* < 0.1.

## References

[1] Machingaidze S., Wiysonge C.S. (2021). Understanding COVID-19 vaccine hesitancy. Nat. Med..

[2] Jaiswal J., LoSchiavo C., Perlman D.C. (2020). Disinformation, misinformation, and inequality-driven mistrust in the time of COVID-19: Lessons unlearned from AIDS denialism. AIDS Behav..

[3] Goldenberg M.J. (2021). Vaccine Hesitancy: Public Trust, Expertise, and the War on Science.

[4] Gao J., Radford B.J. (2021). Death by political party: The relationship between COVID-19 deaths and political party affiliation in the United States. World Med. Health Policy.

[5] Sweileh W.M. (2020). Bibliometric Analysis of Global Scientific Literature on Vaccine Hesitancy in Peer-Reviewed Journals (1990–2019). BMC Public Health.

[6] Palm R., Bolsen T., Kingsland J.T. (2021). The effect of frames on COVID-19 vaccine resistance. Front. Political Sci..

[7] Ophir Y., Jamieson K.H. (2018). Intentions to use a novel Zika vaccine: The effects of misbeliefs about the MMR vaccine and perceptions about Zika. J. Public Health.

[8] Nyhan B., Reifler J., Richey S., Freed G.L. (2014). Effective messages in vaccine promotion: A randomized trial. Pediatrics.

[9] Wakefield A.J. (1999). MMR vaccination and autism. Lancet.

[10] Motta M. (2021). Can a COVID-19 Vaccine Live up to Americans’ Expectations? A Conjoint Analysis of How Vaccine Characteristics Influence Vaccination Intentions. Soc. Sci. Med..

[11] Jennings W., Stoker G., Bunting H., Valgarðsson V.O., Gaskell J., Devine D., McKay L., Mills M.C. (2021). Lack of trust, conspiracy beliefs, and social media use predict COVID-19 vaccine hesitancy. Vaccines.

[12] Van der Weerd W., Timmermans D.R., Beaujean D.J., Oudhoff J., van Steenbergen J.E. (2011). Monitoring the level of government trust, risk perception, and intention of the general public to adopt protective measures during the influenza A (H1N1) pandemic in the Netherlands. BMC Public Health.

[13] Siegrist M., Zingg A. (2014). The role of public trust during pandemics. Eur Psych..

[14] Apuke O.D., Omar B. (2021). Social media affordances and information abundance: Enabling fake news sharing during the COVID-19 health crisis. Health Inform. J..

[15] Gursoy D., Ekinci Y., Can A.S., Murray J.C. (2022). Effectiveness of message framing in changing COVID-19 vaccination intentions: Moderating role of travel desire. Tour. Manag..

[16] Thompson O.P., Ademu L.O., Ademu L.A. (2022). Opium or Elixir? How Adherence to Major World Religions Influence Africans’ Health-Related Behavior During a Pandemic: A Case Study of Nigeria. The Palgrave Handbook of Africa and the Changing Global Order.

[17] Gerend M.A., Shepherd J.E. (2007). Using message framing to promote acceptance of the human papillomavirus vaccine. Health Psychol..

[18] Park S.Y. (2012). The effects of message framing and risk perceptions for HPV vaccine campaigns: Focus on the role of regulatory fit. Health Mark. Q..

[19] Tu Y.C., Lin Y.J., Fan L.W., Tsai T.I., Wang H.H. (2019). Effects of multimedia framed messages on human papillomavirus prevention among adolescents. West. J. Nurs. Res..

[20] Piltch-Loeb R., Savoia E., Goldberg B., Hughes B., Verhey T., Kayyem J., Miller-Idriss C., Testa M. (2021). Examining the effect of information channels on COVID-19 vaccine acceptance. PLoS ONE.

[21] Cacciatore M.A. (2021). Misinformation and public opinion of science and health: Approaches, findings, and future directions. Proc. Natl. Acad. Sci. USA.

[22] Presseau J., Desveaux L., Allen U., Arnason T., Buchan J., Corace K., Dubey V., Evans G.A., Fabrigar L.R., Grimshaw J.M. (2021). Behavioural science principles for supporting COVID-19 vaccine confidence and uptake among Ontario health care workers. Sci. Briefs Ont. COVID-19 Sci. Advis. Table.

[23] Guenther L., Gaertner M., Zeitz J. (2021). Framing as a concept for health communication: A systematic review. Health Commun..

[24] Bullock O.M., Shulman H.C. (2021). Utilizing framing theory to design more effective health messages about tanning behavior among college women. Commun. Stud..

[25] Vaala S.E., Ritter M.B., Palakshappa D. (2022). Framing Effects on US Adults’ Reactions to COVID-19 Public Health Messages: Moderating Role of Source Trust. Am. Behav. Sci..

[26] Unger F., Steul-Fischer M. (2020). The effect of message framing and the presentation of health vs. social consequences on health risk perception. Z. Die Gesamte Versicher..

[27] Van’t Riet J., Ruiter R.A., Werrij M.Q., De Vries H. (2010). Investigating message-framing effects in the context of a tailored intervention promoting physical activity. Health Educ. Res..

[28] Elbert S.P., Ots P. (2018). Reading or listening to a gain-or loss-framed health message: Effects of message framing and communication mode in the context of fruit and vegetable intake. J. Health Commun..

[29] Von Sikorski C., Matthes J., Rossmann C., Hastall M. (2019). Framing-Effekte in Gesundheitsbereich [Framing effects in health communication]. Handbuch der Gesundheitskommunikation [Handbook of Health Communication].

[30] Penţa M.A., Băban A. (2018). Message framing in vaccine communication: A systematic review of published literature. Health Commun..

[31] Poland C.M., Poland G.A. (2011). Vaccine education spectrum disorder: The importance of incorporating psychological and cognitive models into vaccine education. Vaccine.

[32] Zhang Y., Jin Y. (2017). Thematic and episodic framing of depression: How Chinese and American newspapers framed a major public health threat. Athens J. Mass Media Commun..

[33] Druckman J.N. (2004). Political preference formation: Competition, deliberation, and the relevance of framing effects. Am. Political Sci. Rev..

[34] Kahneman D., Tversky A. Prospect theory: An analysis of decision under risk. In *Handbook of the Fundamentals of Financial Decision Making: Part I*; 2013; pp. 99–127. https://www.worldscientific.com/doi/10.1142/9789814417358_0006.

[35] Evans A.M., Krueger J.I. (2009). The psychology (and economics) of trust. Soc. Personal. Psychol. Compass.

[36] Hall M.A. (2006). Researching medical trust in the United States. J. Health Organ. Manag..

[37] Olson O., Berry C., Kumar N. (2020). Addressing parental vaccine hesitancy towards childhood vaccines in the United States: A systematic literature review of communication interventions and strategies. Vaccines.

[38] Idler E.L. (2014). Religion as a Social Determinant of Public Health.

[39] Pargament K.I., Exline J.J., Jones J.W. (2013). APA Handbook of Psychology, Religion, And spirituality (Vol 1): Context, Theory, and Research.

[40] Seddig D., Maskileyson D., Davidov E., Ajzen I., Schmidt P. (2022). Correlates of COVID-19 vaccination intentions: Attitudes, institutional trust, fear, conspiracy beliefs, and vaccine skepticism. Soc. Sci. Med..

[41] Coustasse A., Kimble C., Maxik K. (2021). COVID-19 and vaccine hesitancy: A challenge the United States must overcome. J. Ambul. Care Manag..

[42] Schmid B., Thomas E., Olivier J., Cochrane J.R. (2008). The Contribution of Religious Entities to Health Sub-Saharan Africa [Executive Summary].

[43] Weinberger-Litman S.L., Litman L., Rosen Z., Rosmarin D.H., Rosenzweig C. (2020). A look at the first quarantined community in the USA: Response of religious communal organizations and implications for public health during the COVID-19 pandemic. J. Relig. Health.

[44] Levin J. (2016). Partnerships between the faith-based and medical sectors: Implications for preventive medicine and public health. Prev. Med. Rep..

[45] Dubé E., Laberge C., Guay M., Bramadat P., Roy R., Bettinger J.A. (2013). Vaccine hesitancy: An overview. Hum. Vaccines Immunother..

[46] Puri N., Coomes E.A., Haghbayan H., Gunaratne K. (2020). Social media and vaccine hesitancy: New updates for the era of COVID-19 and globalized infectious diseases. Hum. Vaccines Immunother..

[47] Thunström L., Ashworth M., Finnoff D., Newbold S.C. (2021). Hesitancy toward a COVID-19 vaccine. Ecohealth.

[48] Holton A., Weberling B., Clarke C.E., Smith M.J. (2012). The blame frame: Media attribution of culpability about the MMR–autism vaccination scare. Health Commun..

[49] Leland S., Mohr Z., Piatak J. (2021). Accountability in government contracting arrangements: Experimental analysis of blame attribution across levels of government. Am. Rev. Public Adm..

[50] Piatak J., Mohr Z., Leland S. (2017). Bureaucratic accountability in third-party governance: Experimental evidence of blame attribution during times of budgetary crisis. Public Adm..

[51] Barends A.J., de Vries R.E. (2019). Noncompliant responding: Comparing exclusion criteria in MTurk personality research to improve data quality. Personal. Individ. Differ..

[52] Zack E.S., Kennedy J., Long J.S. (2019). Can nonprobability samples be used for social science research? A cautionary tale. Surv. Res. Methods.

[53] Hydock C. (2018). Assessing and overcoming participant dishonesty in online data collection. Behav. Res. Methods.

[54] Aguinis H., Villamor I., Ramani R.S. (2021). MTurk research: Review & recommendations. J. Manag..

[55] Stritch J.M., Pedersen M.J., Taggart G. (2017). The opportunities and limitations of using Mechanical Turk (Mturk) in public administration and management scholarship. Int. Public Manag. J..

[56] Angrist J.D., Pischke J.S. (2008). Parallel worlds: Fixed effects, differences-in-differences, and panel data. Mostly Harmless Econometrics.

[57] Henderson J., Ward P.R., Tonkin E., Meyer S.B., Pillen H., McCullum D., Toson B., Webb T., Coveney J., Wilson A. (2020). Developing and maintaining public trust during and post-COVID-19: Can we apply a model developed for responding to food scares?. Front. Public Health.

[58] Kuru O., Chan M.P.S., Lu H., Stecula D.A., Jamieson K.H., Albarracín D. (2022). Religious affiliation and philosophical and moral beliefs about vaccines: A longitudinal study. J. Health Psychol..

[59] Iannelli V. Are There Religious Exemptions to Vaccines? 2019. https://www.verywellfamily.com/religious-exemptions-to-vaccines-2633702.

[60] Patrick B. (2020). Studying COVID-19 in light of critical approaches to risk and uncertainty: Research pathways, conceptual tools, and some magic from Mary Douglas. Health Risk Soc..

[61] Sammut G.E., Andreouli E.E., Gaskell G.E., Valsiner J.E. (2015). The Cambridge Handbook of Social Representations.

[62] Sturgis P., Brunton-Smith I., Jackson J. (2021). Trust in science, social consensus, and vaccine confidence. Nat. Hum. Behav..

[63] MacDonald N.E. (2015). Vaccine hesitancy: Definition, scope, and determinants. Vaccine.

[64] Cummings L. (2014). The “trust” heuristic: Arguments from an authority in public health. Health Commun..

[65] Kennedy B., Tyson A., Funk C. (2022). Americans’ Trust in Scientists, Other Groups Declines. https://www.pewresearch.org/science/2022/02/15/americans-trust-in-scientists-other-groups-declines/.

[66] Mede N.G., Schäfer M.S. (2022). Science-related populism declining during the COVID-19 pandemic: A panel survey of the Swiss population before and after the Coronavirus outbreak. Public Underst. Sci..

[67] Wintterlin F., Hendriks F., Mede N.G., Bromme R., Metag J., Schäfer M.S. (2022). Predicting Public Trust in Science: The Role of Basic Orientations Toward Science, Perceived Trustworthiness of Scientists, and Experiences with Science. Front. Commun..

[68] O’Brien T.C., Palmer R., Albarracin D. (2021). Misplaced trust: When trust in science fosters a belief in pseudoscience and the benefits of critical evaluation. J. Exp. Soc. Psychol..

[69] Merkley E., Loewen P.J. (2021). Anti-intellectualism and the mass public’s response to the COVID-19 pandemic. Nat. Hum. Behav..

[70] Sulik J., Deroy O., Dezecache G., Newson M., Zhao Y., El Zein M., Tunçgenç B. (2021). Trust in Science Boosts Approval, but Not Following COVID-19 Rules. https://scholarworks.iupui.edu/handle/1805/25719.

[71] Gross K. The limits of framing: How framing effects may be limited or enhanced by individual-level predispositions. Proceedings of the Annual Meeting of the Midwest Political Science Association.

[72] Brewer P.R. (2001). Value words and lizard brains: Do citizens deliberate about appeals to their core values?. Political Psychol..

[73] Chong D., Druckman J.N. (2007). Framing theory. Annu. Rev. Polit. Sci..

[74] Druckman J.N., Nelson K.R. (2003). Framing and deliberation. Am. J. Polit. Sci..

[75] Lin C., Tu P., Beitsch L.M. (2020). Confidence and receptivity for COVID-19 vaccines: A rapid systematic review. Vaccines.

[76] Callaghan T., Moghtaderi A., Lueck J.A., Hotez P.J., Strych U., Dor A., Franklin Fowler E., Motta M. (2020). Correlates and disparities of COVID-19 vaccine hesitancy. Available at SSRN 3667971. https://www.vaccineacceptance.org/correlates-and-disparities-of-covid-19-vaccine-hesitancy/.

[77] Peretti-Watel P., Seror V., Cortaredona S., Launay O., Raude J., Verger P., Fressard L., Beck F., Legleye S., l’Haridon O. (2020). A future vaccination campaign against COVID-19 at risk of vaccine hesitancy and politicization. Lancet Infect. Dis..

[78] Soares P., Rocha J.V., Moniz M., Gama A., Laires P.A., Pedro A.R., Dias S., Leite A., Nunes C. (2021). Factors associated with COVID-19 vaccine hesitancy. Vaccines.

[79] Ruisch B.C., Moore C., Granados Samayoa J., Boggs S., Ladanyi J., Fazio R. (2021). Examining the left-right divide through the lens of a global crisis: Ideological Differences and their implications for responses to the COVID-19 pandemic. Political Psychol..

[80] Krämer B., Klingler M. (2020). A bad political climate for climate research and trouble for gender studies: Right-wing populism as a challenge to science communication. Perspect. Popul. Media Ave. Res..

[81] Murphy K. (2005). Regulating more effectively: The relationship between procedural justice, legitimacy, and tax non-compliance. J. Law Soc..

[82] Scholz J.T., Braithwaite V., Levi M. (1998). Trust, taxes, and compliance. Trust and Governance.

[83] Chanley V.A., Rudolph T.J., Rahn W.M. (2000). The origins and consequences of public trust in government: A time series analysis. Public Opin. Q..

